# Study on the Hydrophobic Modification of MTES/NH_3_ Vapor Surface Treatment for SiO_2_ Broadband Anti-Reflection Coating

**DOI:** 10.3390/ma15030912

**Published:** 2022-01-25

**Authors:** Qianyang Fan, Hanxi Liu, Xinli Jia, Lianghong Yan, Bo Jiang

**Affiliations:** 1 College of Chemistry, Sichuan University, Chengdu 610064, China; Fanqianyang@stu.scu.edu.cn (Q.F.); liangbuzhi@foxmail.com (H.L.); jiaxinli@stu.scu.edu.cn (X.J.); 2 Research Center of Laser Fusion, China Academy of Engineering Physical, Mianyang 621900, China; yanlianghong@126.com

**Keywords:** vapor treatment, hydrophobic, thickness, broadband anti-reflection

## Abstract

In this article, ammonia and methyltriethoxysilane (MTES) were chosen as vapor phase modifiers for the base-catalyzed SiO_2_ film. The surface of the film became more dense because of the hydroxyl condensation under the catalyst of ammonia, while the introduction of methyl groups by MTES of vapor treatment hindered the condensation to avoid over-change in film thickness. The hydrophobic of film was improved while the surface roughness of the film increased after treatment. The treated double-layer broadband anti-reflection (AR) coating retains high optical properties with the transmittance of 99.61%, 98.85%, and 99.16% at 355 nm, 532 nm, and 1064 nm, respectively. After exposing to the high humidity condition for 30 days, the broadband AR coating after treatment shows good optical durability, and the transmittance at 355 nm only drops by 0.12%. This vapor surface treatment can find potential application in high-power laser systems and solar cells.

## 1. Introduction

Anti-reflection coating is usually applied to the surface of optical devices to reduce the energy loss caused by light reflecting [1]. Many methods have been exploited for the preparation of AR coatings such as phase separation, etching, physical vapor deposition, and the sol–gel method [2,3,4,5,6]. Among these methods, the sol–gel method is widely used due to its mild preparation conditions, simple operation process, and low cost. A sol–gel-derived silica AR coating can effectively reduce the loss of light energy with high laser damage threshold (LIDT) [7]. The AR coating tends to absorb moisture and contaminants in the surrounding owing to the considerable hydroxyl groups on the surface of the coating [8]. As a result, the refractive index and thickness of the film will be changed, affecting the original optical properties during application. Improvement of the stability of the AR coating is a key factor for the practical application of the coating.

Vapor phase treatment is often used to improve the optical stability of the anti-reflective coating [9]. Ammonia is used to modify the film under vapor phase for the first time, and the treatment provides a better coating abrasion resistance with a higher LIDT [10]. It has already been demonstrated that the hydroxyl group is involved in increasing the densification of the films [11]. A hydrophobic AR film on polymer substrates is obtained by the sol–gel method with an ammonia vapor treatment, and it shows the mechanism of Ostwald-ripening-type structural modification under vapor treatment [12]. Then, ammonia is used with organic silane to modify film in the later study, an AR coating with good stability in different environment is obtained by the combined vapor phase surface treatment [13,14]. The vapor treatment process is simple to implement and can be carried out at room temperature and atmospheric pressure with minimal equipment requirements and minimal substrate handling [15]. However, the thickness of the film often changes markedly after vapor treatment [16], which is of no concern in single-layer film but is critical for a multi-layer broadband AR system that requires precise control of film thickness [17]. The vapor treatment in broadband AR film is hardly studied, while the ability to maintain the optical property in a variety of tough environments might be useful and beneficial. To accomplish that, a method in which the stability of the broadband film can be enhanced while maintaining the thickness would be of great interest.

In our work, we propose a method for the surface treatment of the silica film by using ammonia and MTES in vapor phase at above 100 °C for 2 h (MTES/NH_3_ vapor treatment). The thickness of the film obtained by the co-precursors of MTES and tetraethoxysilane (TEOS) is basically unchanged after treatment, while the hydrophobic of film increased a lot, especially at a low M/T ratio. The double-layer AR coating after treatment retained high transmittance property with better stability in a humid environment. This surface treatment may be of great significance to enhance and optimize the multi-layer film broadband anti-reflection systems, which require precise thickness to maintain the film.

## 2. Materials and Methods

The sols were prepared via the Stöber method using TEOS and MTES as co-precursors, ammonia as a catalyst, and ethanol as a solvent. Precursors were purchased from TCI (Tokyo, Japan), and the remaining reagents was purchased from Greagent (Shanghai, China). The molar ratios of (TEOS + MTES): EtOH: H_2_O: NH_4_OH was 1:37.6:3.25:0.17. The mixture was stirred at 30 °C for 2 h and then aged at 25 °C for 12 days. The molar ratio of MTES to TEOS was 0, 0.1, 0.3, 0.5, and 1, respectively. The ratio of 0.3 was prepared for the top layer of a double-layer AR coating.

The composite sol for the bottom film was obtained by mixing the acid/base catalyzed sols according to the mass ratio of 1:4.25 [18]. The acid-catalyzed SiO_2_ sol was prepared by mixing a solution of TEOS, EtOH, H_2_O, and HCl. The molar ratio of TEOS:EtOH:H_2_O:HCl was 1:36.8:4.01:4.16 × 10^−3^. The base-catalyzed SiO_2_ sol was prepared by mixing a solution of TEOS, EtOH, H_2_O, NH_3_·H_2_O, and the ratio of TEOS:EtOH:H_2_O:NH_4_OH was 1:37.5:2.78:0.3125, respectively. The sols were stirred at 30 °C for 2 h and aged in a closed glass container at 25 °C for 7 days.

The sols were deposited on well-cleaned Fused silica glasses by dip coating to form AR coatings. The substrate was immersed vertically in the constant solid suspension, and the optical thickness of the film is controlled by varying the deposition rate. The double-layer film was obtained by depositing sequentially in the sols. All the film was heated in 160 °C for 1 h to stabilize the structure before the test. The as-deposited coatings were set in a sealed container with ammonia 3 g and MTES 3 g inside. The container was heated to 100 °C for 2 h and then raised to 160 °C for 1 h to remove the residual chemicals and reinforce the film. The film after treatment was well-prepared after cooling down to room temperature.

The transmission spectra were collected with a UV–visible spectrometer (PerkinElmer Lambda 750, Waltham, MA, USA). The refractive index of these coatings was measured using spectroscopic ellipsometry at λ = 633 nm (HORIBA UVISEL Kyoto, Japan). The morphology of the coating surface was observed using AFM (SEIKO SPA-400 Tokyo, Japan). The cross-sectional morphology of the double-layer coating was measured by SEM (Hitachi S4800 Japan Chiyoda, Tokyo, Japan). The chemical properties of the coating were investigated through FTIR (Bruker Tensor 27, Karlsruhe, Germany). The water contact angles of the silica coating were measured using a JGW-360B contact angle goniometer (Chengde, China) at ambient temperature (error bar ≤ 1°).

## 3. Results and Discussion

### 3.1. The Optical Performance and Surface Microstructure of Single-Layer Film after Treatment

**Figure 1:**
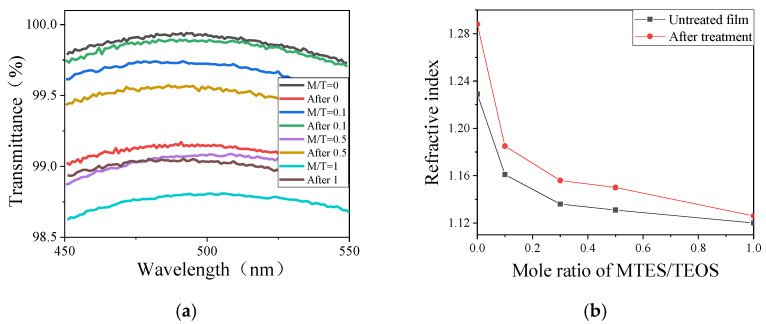
The transmittance spectra (**a**) and refractive index (**b**) of a series molar ratio of M/T film and the film after treatment.

MTES and TEOS were used as co-precursors to obtain sols with a low refractive index [19]. The transmittance of the varied MTES/TEOS (M/T) mole ratio film and the resultant film after treatment are shown in Figure 1a. It can be found that the central wavelength for each M/T mole ratio remains the same after treatment. Figure 1b shows the change of refractive index of film before and after treatment as a function of the M/T ratio. With the increase in M/T ratio, the refractive index of untreated film decreased rapidly at first; then, it slowed down and approached a minimum value of 1.12. In addition, the refractive index of film increased after treatment, and the extent of the increase decreased gradually with the increase in M/T molar ratio. The interpretation of such results could be done in the following way. The surface of the film with a high M/T ratio has more methyl groups; the remaining hydroxyl groups on the surface would react with the adjacent particles and be replaced by the methyl after vapor treatment. The higher the M/T ratio of film, the fewer hydroxyl groups on the surface and the weaker the effort on the film by the MTES/NH_3_ treatment.

The thickness of the film can be calculated by the formula of the single-layer quarter-wavelength (λ/4) AR coating about the thickness (d), refractive index (n), and wavelength (λ):

(1) 4nd=λ

**Figure 2:**
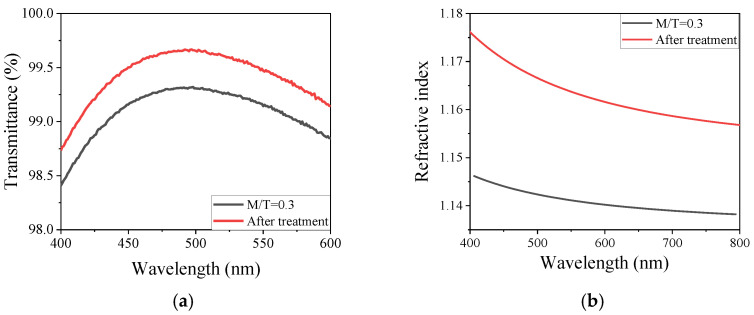
The transmittance spectra (**a**) and refractive index (**b**) of untreated film (M/T = 0.3) and the film after treatment.

Figure 2a shows the transmittance spectrum of coatings derived from the sol with M/T = 0.3, the maximum transmittance of the film was raised from 99.32% to 99.66% with the central wavelength maintained at 498 nm, and the corresponding refractive index of the film was changed from 1.14 to 1.16, as shown in Figure 2b. The calculated thickness of the film before and after treatment are 109 nm and 107 nm, respectively. Table 1 shows the detailed parameters of the M/T ratio film before and after treatment. It can be observed that the thickness of film used to be 2–4 nm thinner after treatment, while it can be seen as basically unchanged. That results indicate that a precise control of the thickness of the film is realized by the MTES/NH_3_ vapor treatment.

**Figure 3:**
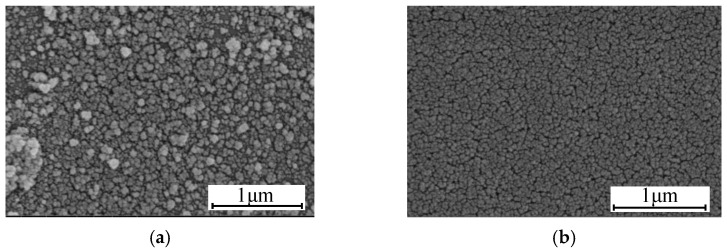
The surface morphologies by SEM of unmodified (M/T = 0.3) film (**a**) and the film after treatment (**b**).

**Figure 4:**
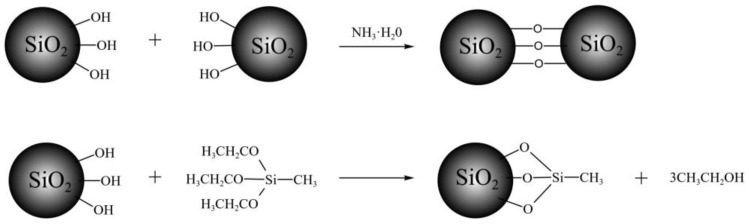
Schematic diagram of chemical reactions between SiO_2_ film and MTES/NH_3_.

It is well known that the presence of ammonia can be a catalyst to the condensation of hydroxyl groups with the adjacent silanol group on the surface of AR coating [20]. It was clear that the surface porosity of the film is reduced and the appearance of the film show more densely after the vapor phase treatment, as shown in Figure 3. While it was consistent with the result of the change of the refractive index that the lower the porosity, the higher the refractive index, according to the relationship between surface porosity and refractive index:

(2) $$n_{p}^{2}=(n_{d}^{2}-1)(1-P)+1$$

where $n_{p}$ and $n_{d}$ represent the refractive index of porous and dense material respectively, and P is the porosity of the film. As for the mechanism for the thickness retained of the above films, we speculated that the surface hydroxyl groups of the film react with the adjacent silanol group and react with MTES simultaneously in the vapor treatment of MTES/NH_3_. A schematic diagram of chemical reactions between the base-catalyzed SiO_2_ film and MTES/NH_3_ is shown in Figure 4. The condensation of silanol groups gave rise to coating shrinkage, while the introduction of methyl groups hindered the condensation because of the steric hindrance effect and produced more pores in the resultant film. These two competitive reactions reached a balance in thickness, resulting in the retaining of film thickness after the treatment of MTES/NH_3_.

### 3.2. The Hydrophobic Properties of the Film after Treatment

**Figure 5:**
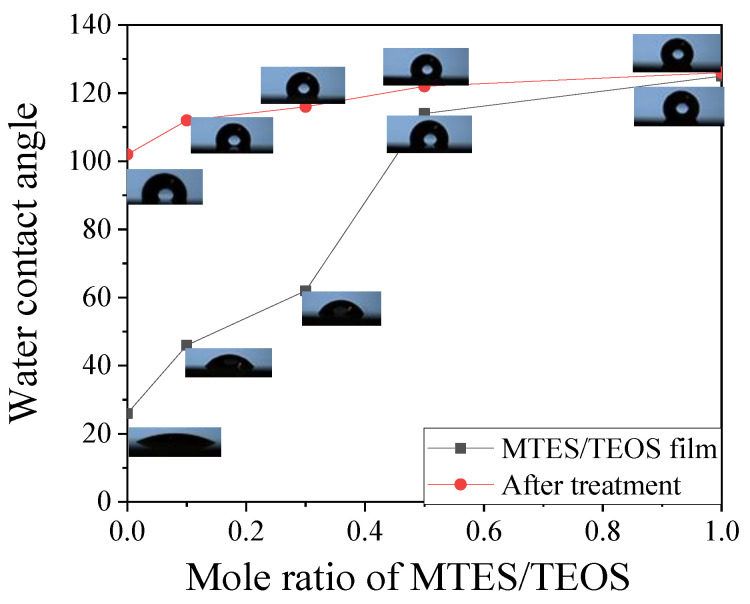
The water contact angle (WCA) of untreated film and the film after treatment.

**Figure 6:**
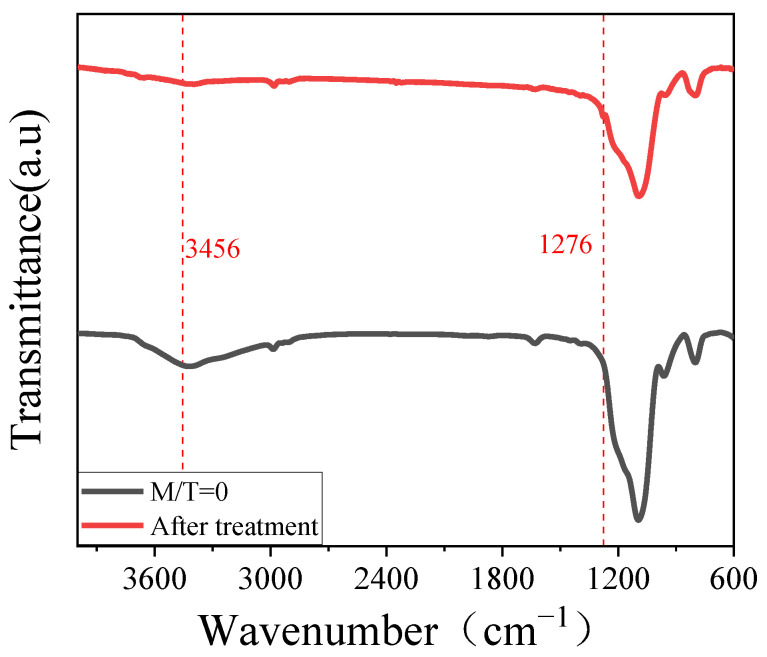
The FTIR spectra of unmodified (M/T = 0) film and the film after MTES/NH_3_ vapor treatment.

Figure 5 shows the change of water contact angle of silica coating of MTES/TEOS film before and after treatment, each value is the average of six measurements. It can be seen that the WCA of the coating increased with the increasing of MTES content, and the WCA of the M/T film increased after treatment especially when the ratio of M/T < 0.5. The surface wettability of the anti-reflection film is determined by its chemical composition and surface microstructure. Figure 6 shows the FTIR spectra of M/T = 0 and resultant film after treatment. The absorption band at 3458 cm^−1^ is attributed to the hydroxyl group, and the intensity of the absorption band at 3458 cm^−1^ decreases dramatically after treatment. There appears a new absorption band at 1276 cm^−1^ after treatment, corresponding to stretching vibrations of Si-CH_3_, which is the direct evidence of modification.

**Figure 7:**
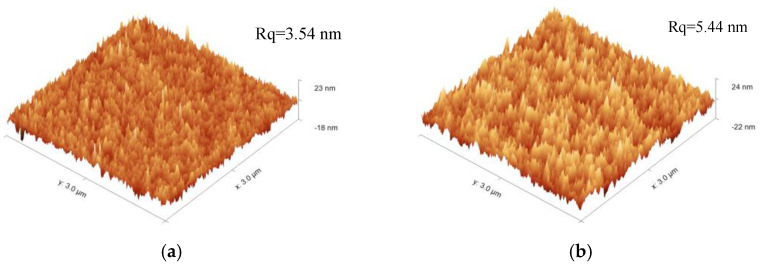
The surface morphologies by AFM of untreated (M/T = 0.3) film (**a**) and the film after treatment (**b**).

In addition to the chemical composition, the microstructure plays an important role in the wettability of a surface, and it can be expressed by Wenzel’s equation [21]:

(3) cosθm=rcosθ

where θm is the contact angle on the rough surface, r is the apparent roughness coefficient (r > 1), and θ is the Young’s contact angle. The contact angle of the film increases with the increase in the roughness of the film, while the film is a rough hydrophobic surface. Figure 7 represents the surface morphologies of the film (M/T = 0.3) by AFM. The roughness of the film increased after treatment, the root-mean-square roughness (Rq) of the untreated film and the film after treatment were 3.54 nm and 5.44 nm, respectively. The increase in roughness and the change of the surface chemical composition improved markedly the film’s hydrophobicity.

### 3.3. Application of the MTES/NH_3_ Vapor Treatment for Double-Layer Tri-Wavelength AR Coating

TF Calc^TM^ (Thin Film Design Software Version 3.5) was used to simulate the refractive index and thickness to match the double-layer film with tri-wavelength anti-reflection. The refractive index of the top film was 1.14 and the bottom was 1.26. The optimum central wavelengths of films were set to be quarter-wave at 480 nm, and the thickness of the top and bottom film were 105 nm and 95 nm, respectively. The top layer with a low refractive index of 1.14 was prepared by a base-catalyzed sol–gel process using MTES and TEOS as co-precursors, while the bottom layer was prepared by the hybridization of acid-catalyzed and base-catalyzed SiO_2_ sols. The thickness of each layer is controlled by varying the deposition rate to meet the requirement.

**Figure 8:**
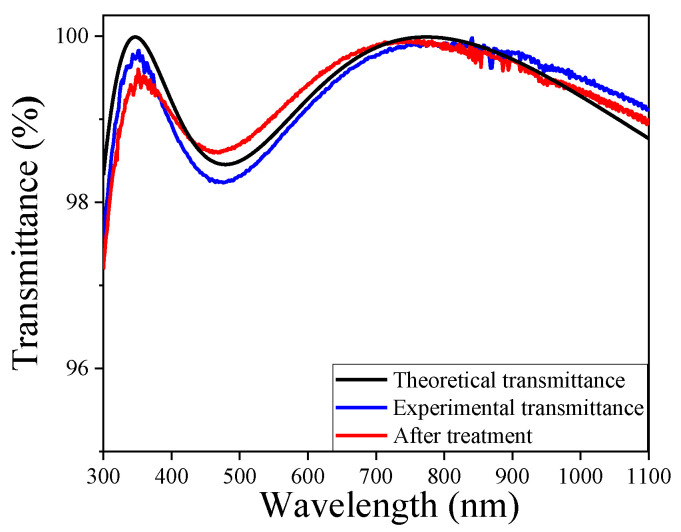
The transmittance spectra of double-layer tri-wavelength AR coating.

Figure 8 shows the theoretical AR coating with nearly 100%, 98.0%, and 99.0% at 355 nm, 532 nm, and 1064 nm, respectively. The experimental transmittance of film is 99.85% at 355 nm, while it reached 98.49% and 99.31% at 532 nm and 1064 nm, respectively. The decrease in the short-wavelength regions was related to the roughness of the film, which increases the scattering of light. After the film is modified, the film had slightly changed in terms of transmittance with a maximum transmittance of 99.61% at 355 nm. The AR film after treatment had excellent anti-reflection performance and showed 98.85% and 99.16% at 532 nm and 1064 nm, respectively. The spectra of transmittance revealed that the high anti-reflection performance of the film could be kept after the vapor treatment of MTES/NH_3_.

**Figure 9:**
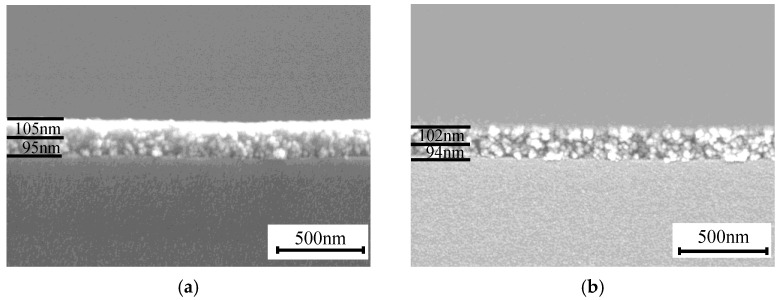
Cross-sectional SEM image of the untreated double-layer AR coating (**a**) and the film after treatment (**b**).

The cross-section morphologies of the double-layer film are shown in Figure 9, the thickness of the untreated double-layer film was 105 nm and 95 nm, and the thickness was barely changed while the thickness of the treated film was 102 nm and 94 nm, respectively, which is consistent with previous assumptions.

### 3.4. Environmental Stability of Broadband Double-Layer AR Film

**Figure 10:**
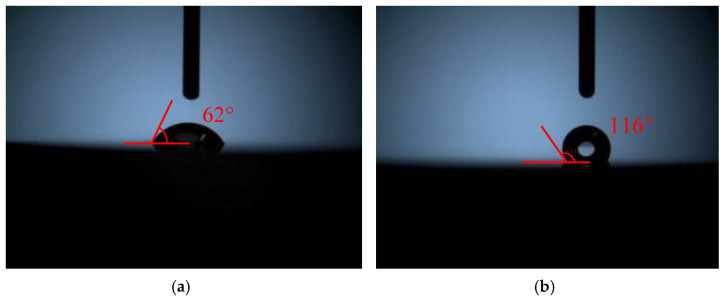
The water contact angle of untreated (**a**) double-layer broadband film and the film after treatment (**b**).

**Figure 11:**
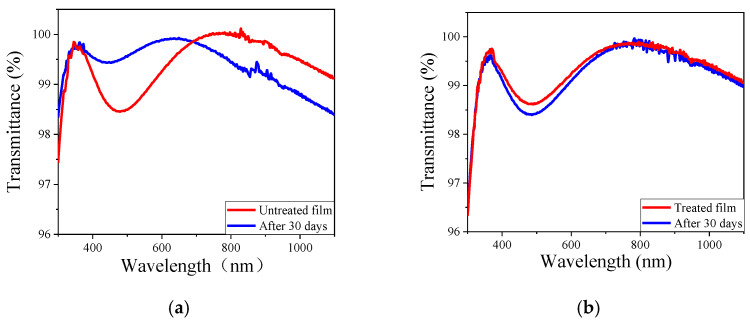
Transmittance spectrum of (**a**) untreated double-layer film and the film after being exposed in humid environment; (**b**) transmittance spectra of treated film after exposing to humid environment.

Hydrophobicity is a key factor of the coating that directly affects its optical stability in humidity conditions. The WCA of the double-layer broadband film was largely increased from 62° to 116° after MTES/NH_3_ vapor treatment, as shown in Figure 10. In order to further reveal the effect of modification on the stability of film, the film was placed in a closed environment with a temperature of 25 °C and humidity of 99% for a certain period of time. The deviations of transmittance spectrum of the film before and after being exposed to the humid environment are shown in Figure 11. The shape and transmittance of the untreated film changed dramatically after the exposing time up to 30 days, and the transmittance dropped below 99.00% above 800 nm. The film after MTES/NH_3_ vapor treatment still maintained its high transmittance, only a decrease of 0.12%, 0.19%, and 0.09% was observed for the transmittance value at 355 nm, 532 nm, and 1064 nm, respectively. Hence, MTES/NH_3_ vapor treatment afforded the double-layer film after a better optical stability in high humidity environment.

## 4. Conclusions

In summary, MTES/NH_3_ vapor treatment is a very suitable method for the surface modification of the silicate AR coating.

♦ The hydrophobic performance of the film after treatment can be greatly enhanced while the thickness of the film was kept almost unchanged, which is very useful for a multi-layer AR coating system.
♦ By this method, a hydrophobic double-layer tri-wavelength broadband AR coating was obtained with a high transmittance value of 99.60%, 98.85%, and 99.16% at 355 nm, 532 nm, and 1064 nm, respectively.
♦ Moreover, the treated coating exhibited a good optical stability in humidity environment.

Therefore, we believe that the MTES/NH_3_ vapor treatment method may have important application for the optimization of multi-layer AR coating systems that require precise thickness control.

## Figures and Tables

**Table 1:** Changes in the refractive index and thickness of M/T ratio film after vapor treatment.

M/T Ratio	Refractive Index (*n*)		Thickness (*d*)	
	Before Treatment	After Treatment	Before Treatment	After Treatment
0	1.22	1.28	100	96
0.1	1.16	1.18	104	102
0.3	1.14	1.16	109	107
0.5	1.13	1.15	111	108
1	1.12	1.12	112	100

## Data Availability

The data presented in this study are available upon request.
